# Estimation of Total Cost Required in Controlling COVID-19 Outbreaks by Financial Incentives

**DOI:** 10.3390/ijerph20021217

**Published:** 2023-01-10

**Authors:** Sangkwon Kim, Youngjin Hwang, Chaeyoung Lee, Soobin Kwak, Junseok Kim

**Affiliations:** Department of Mathematics, Korea University, Seoul 02841, Republic of Korea

**Keywords:** COVID-19, Monte Carlo simulation, financial incentives, policymakers

## Abstract

In this article, we present a Monte Carlo simulation (MCS) to estimate the total cost required to control the spread of the COVID-19 pandemic by financial incentives. One of the greatest difficulties in controlling the spread of the COVID-19 pandemic is that most infected people are not identified and can transmit the virus to other people. Therefore, there is an urgent need to rapidly identify and isolate the infected people to avoid the further spread of COVID-19. To achieve this, we can consider providing a financial incentive for the people who voluntarily take the COVID-19 test and test positive. To prevent the abuse of the financial incentive policy, several conditions should be satisfied to receive the incentive. For example, an incentive is offered only if the recipients know who infected them. Based on the data obtained from epidemiological investigations, we calculated an estimated total cost of financial incentives for the policy by generating various possible infection routes using the estimated parameters and MCS. These results would help public health policymakers implement the proposed method to prevent the spread of the COVID-19 pandemic. In addition, the incentive policy can support various preparations such as hospital bed preparation, vaccine development, and so forth.

## 1. Introduction

The outbreak of the coronavirus disease 2019 (COVID-19) pandemic is the most formidable challenge that humanity has witnessed in this century [1]. Many preventive measures have proven effective against the COVID-19 pandemic such as wearing masks, self-quarantine, isolation, lockdown, travel restrictions, social distancing, school closures, high-risk business restrictions, and vaccination. In particular, wearing face masks has a significant protective effect against COVID-19 [2,3]. Social distancing reduces the incidence of the disease by increasing the distance between people [4,5,6]. As a strong social distancing measure, school closures were temporarily employed to reduce contact among children [7]. Several countries have limited the operations of high-risk businesses such as restaurants, bars, and gymnasiums during prior COVID-19 outbreaks [8]. If contact-tracing systems are improved to monitor people’s daily contact information, close contacts of COVID-19 patients can be identified. Baker et al. [9] suggested a strategy to minimize disease transmission by studying an optimal quota of COVID-19 tests using contact information. Vaccination is one of the most effective medical interventions to significantly decrease the spread of infectious diseases [10,11,12,13]. However, vaccination hesitancy is a major global problem associated with COVID-19. To improve vaccine uptake, educating people regarding the safety, effectiveness, and rigorous testing and evaluation of existing vaccines is important [14]. More than 70% of the population must be inoculated to achieve herd immunity. For details regarding vaccination, please refer to previous studies [15,16]. Fallucchi et al. [17] investigated and analyzed behavioral factors related to individual willingness-to-test (WTT) using behavioral and economic insights to increase voluntary participation in COVID-19 testing. In [18], the authors presented the results of a systematic literature review on the effects of economic incentives on participation rate in prevention management to reduce the risk of diseases such as cancer.

The main purpose of this study is to present a Monte Carlo simulation (MCS) conducted to estimate the total cost required to control the spread of the COVID-19 pandemic through financial incentives. We present various financial incentive policies and analyze them in terms of cost to encourage voluntary COVID-19 testing of unidentified infected people. It should be noted that the proposed method can also be applied in the future in response to new global epidemic outbreaks by delaying the spread of an epidemic outbreak to ensure sufficient time for vaccine development. We provide the Matalabe code for the simulation main code in Appendix A.

## 2. Literature Review

In this section, we briefly review the relevant literature and highlight the novelty of the proposed methodology compared to previous approaches. If contact-tracing data for all populations can be obtained, a contact-tracing system will be able to end the COVID-19 pandemic. However, contact tracing data may violate an individual’s privacy. To motivate the program users to provide more contact-tracing data, an incentive algorithm has been developed [19]. Munzert et al. [20] showed that a financial incentive to use digital contact tracking apps is an effective measure to contain the COVID-19 pandemic. However, despite regular reminders and financial incentives, study participants showed low response rates [21]. In this study, we focus on finding the links in the chain of transmission between infected people and quickly identifying and isolating unidentified infected people rather than requiring sensitive contact-tracing data from people. The proposed method can motivate people to voluntarily take a COVID-19 test and receive a financial incentive by providing information such as the person from whom they contracted the infection if they tested positive for COVID-19. Thus, infected people are given a choice as to whether to provide sensitive contact-tracing data, and they only have to provide a fraction of the overall contact-tracing data collected on their movements.

In [22], the authors assessed the causes of stress for front-line healthcare professionals and the impact of protective measures applied to counteract stress. It was also reported that protective measures such as salary incentives, encouragement to work in teams, and support from senior management can relieve stress, highlighting the importance of appropriate protective measures. Our proposed method can help reduce stress for healthcare professionals with simple tasks. For example, more financial incentives can be provided if an individual makes an appointment with a healthcare professional in advance, or incentives can be adjusted based on the opinion of healthcare professionals to help reduce healthcare professional stress.

As described in [23], Israel has provided incentives for vaccination to protect the health of all people from the COVID-19 pandemic. Laws and regulations enacted as vaccine incentives during the pandemic include negative incentives to limit the freedom of many people, including Green Passes. Although this can ultimately have positive effects, many people may object to such measures. Because most vaccines require two or more injections spaced three to four weeks apart, based on the empirical literature, studies have recommended delivering some financial incentives after the first injection and more financial incentive after the second injection to promote high levels of adherence to COVID-19 vaccines [24]. The key issue is how to pay financial incentives. Recent studies have shown that a lottery can improve COVID-19 vaccine injection more than a lump-sum payment based on experiments in a virtual environment [25]. See the studies [25,26,27] for methods to provide financial incentives in the form of a lottery during the COVID-19 pandemic. Although vaccines are highly effective, they have not been able to fully protect people from the threat of COVID-19. It may take longer to build herd immunity because people refuse or hesitate to get the COVID-19 vaccine for a variety of reasons [28]. Moreover, the effectiveness of the vaccine is reduced against the COVID-19 variants [29]. Compared to the previous methods, the main novelty of the proposed algorithm is that it can estimate the total cost required in controlling the spread of a new epidemic pandemic by financial incentives under various incentive policies. It should noted that the proposed method can be useful in the early times for combating new global epidemic outbreaks by delaying the spread of the epidemic outbreak to ensure enough time for vaccine development.

## 3. Methods

Now, we present the proposed algorithm for estimating the total cost required to control the spread of the COVID-19 pandemic through financial incentives after generating an infection route using MCS with the given confirmed data. Table 1 lists the descriptions of the parameters used in the proposed algorithm.

### 3.1. Infection Networks

We define the individual-specific information $C_{m}^{p}$ of infected people during the observation period. Here, *C* denotes the confirmed person, and *p* is the infectious person identification number of a person that infected the confirmed COVID-19-positive person *m*. Here, *m* is the number assigned to identify the infected, such as a social security number or passport number, which is simply used as a number assigned according to the order of confirmation. For example, $C_{7}^{2}$ indicates that it was identified seventh during the observation days and was infected by the second confirmed person. Individual-specific information can be found through epidemiological investigations. If the infection route cannot be found, we set it to p=0.

**Figure 1:**
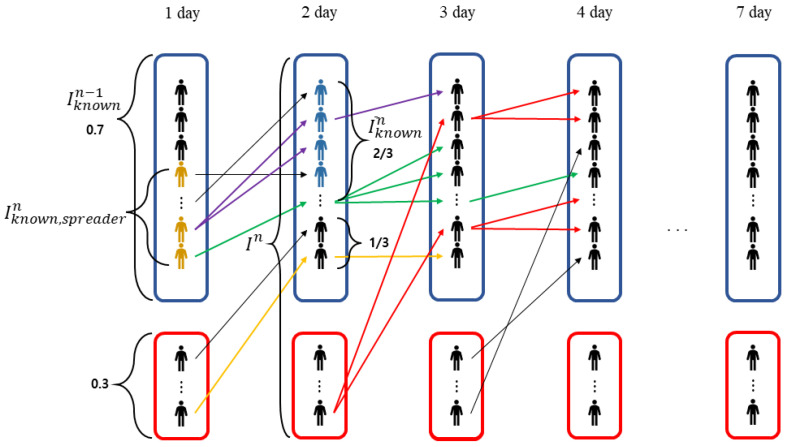
Schematic illustration of the infection routes between the confirmed people. On the first day, people shown in yellow are confirmed as being infected, who infect people the next day. Here, $r_{1}=0.7$, which is the ratio of people in the blue box to the total number of people on day 1, and $r_{2}=0.3$, which is the ratio of people in the red box to the total number of people on day 1.

In the simulation, the infection route is generated by applying the MCS. Each day, $r_{1}$ is the ratio of confirmed groups whose infection route is identified to the total number of confirmed COVID-19-positive people; that is, $N_{n}:C\left(n\right)$. $r_{2}$ is the ratio of the group infected from the confirmed group whose infection route was identified on the previous day to the confirmed group whose infection route was identified. Figure 1 shows a schematic diagram of the infection routes among the confirmed people during the observation period (7 days). In Figure 1, the confirmed people in the blue and red boxes are a group of confirmed people who know and do not know their parent contacts, respectively. The arrow indicates the route of infection, and the color of the arrow indicates different chains. Here, the ratios $r_{1}$ and $r_{2}$ are 7/10 and 2/3, respectively. Finally, in Figure 1, the people indicated in yellow are those who infected someone the next day, among those whose infection route was confirmed, $I_{known,spreader}^{n}$, and the people shown in blue are the those who are infected by someone who knows the infection route, ${\bar{I}}_{known}^{n}$.

The main algorithm for the infection network between confirmed cases is given as follows:

*Step (1)*  *Generates the identification number set $I_{n}$ using data of the number of confirmed cases on the n-th day.*
*Step (2)*  *Generates the identification number set of people who know their parents $I_{known}^{n}$ by random sampling without replacement by the ratio $r_{1}$ from $I_{n}$.*
  *$I_{known}^{n}=\left\{m:C_{m}^{p}\;st\;p\neq 0\;for\;m\in I_{n}\right\}$ and $n\left(I_{known}^{n}\right)=\left[r_{1}n\left(I_{n}\right)\right]$ where n(·) is the number of elements in the set and [x] is the greatest integer not greater than x.*
*Step (3)*  *Using the ratio $r_{2}$, randomly sample ${\bar{I}}_{known}^{n}$ from $I_{known}^{n}$ without replacement.*
*Step (4)*  *Using $n({\bar{I}}_{known}^{n})$, randomly sample $I_{known,spreader}^{n}$ from $I_{known}^{n-1}$ and using $n(I_{unknown}^{n}\backslash {\bar{I}}_{known}^{n})$, randomly sample $I_{known,spreader}^{n}$ from $I_{known}^{n-1}$ with replacement.*
*Step (5)*  *Randomly match $(I_{spreader}^{n}$ and ${\bar{I}}_{known}^{n})$ and $(I_{unknown,spreader}^{n}$ and $I_{unknown}^{n}\backslash {\bar{I}}_{known}^{n})$.*

**Figure 2:**
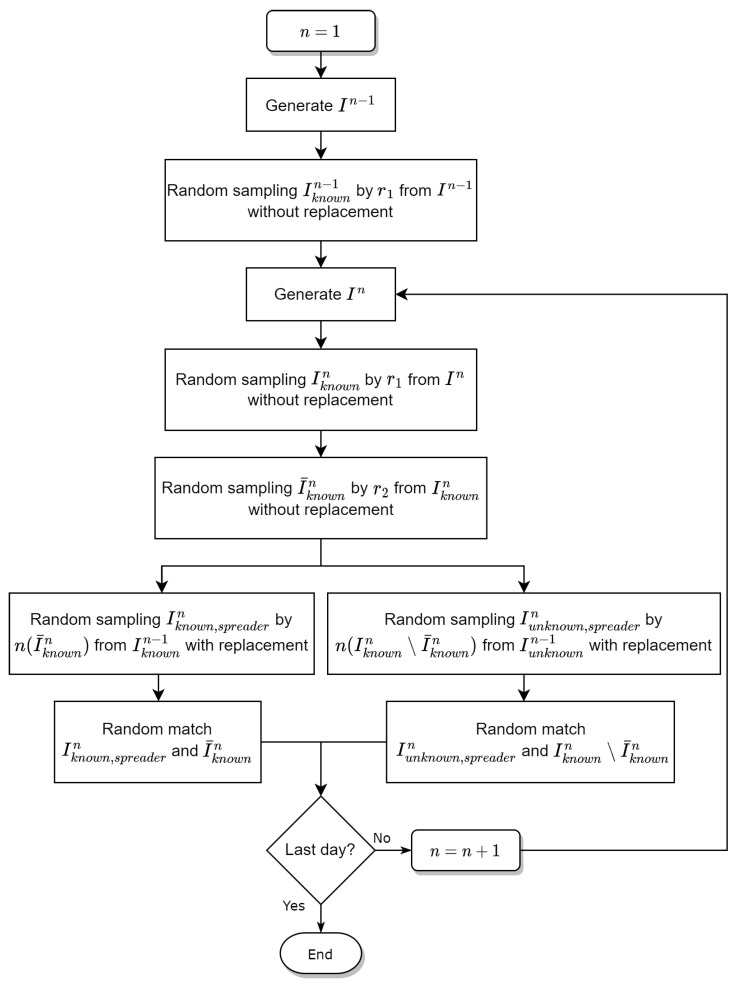
Diagram of the algorithm that generates the infected network.

We repeated the algorithm to build an infection network with information of the confirmed people during the observation days and tracked individual infection routes for patients identified on the last day; see Figure 2.

**Figure 3:**
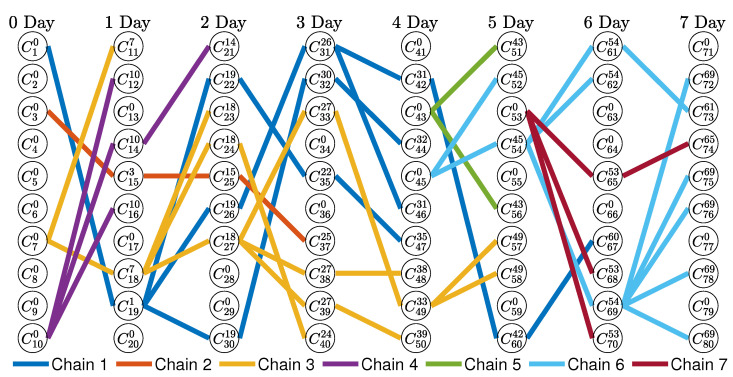
Infection network during 7 days. Here, C(n)=10 for n=0,1,⋯,7. The superscript of infected person *C* is the number of the parent who infected the patient *C*, and the subscript is the unique number of the infected person *C*. The number of chains is 7, and one chain is expressed with the same color.

Figure 3 shows the infection network generated using the proposed algorithm when the observation period was 7 days and there were 10 confirmed cases every day. The number of chains is 7, which was expressed in seven colors in Figure 3.

### 3.2. Incentive Policies

To prevent the abuse of financial incentive policy, several conditions should be satisfied to receive the incentive. Let us consider some possible policies:

- P1: Compensate every confirmed people equally by $U_{P}$.
- P2: Compensate by $U_{C}$ per chain and distribute equally to those who belong to each chain.
- P3: Compensate by $U_{C}$ per chain and distribute unevenly (reasonably) to those who belong to each chain.

In the case of P1, the government can provide economic help to confirmed people by simply paying the same compensation to all confirmed people. Then, the government needs $R=U_{P}\times N$ to realize the P1 policy, where $U_{P}$ is a unit of reward per person for individual payments, and *N* is the total number of confirmed infected people during the observation days. The P2 and P3 policies may not only help economically but also support the government’s efforts to encourage suspected people who had symptoms to voluntarily take the COVID-19 test. These policies can help control the transmission of coronavirus by improving the quality of epidemiological investigations.

**Figure 4:**
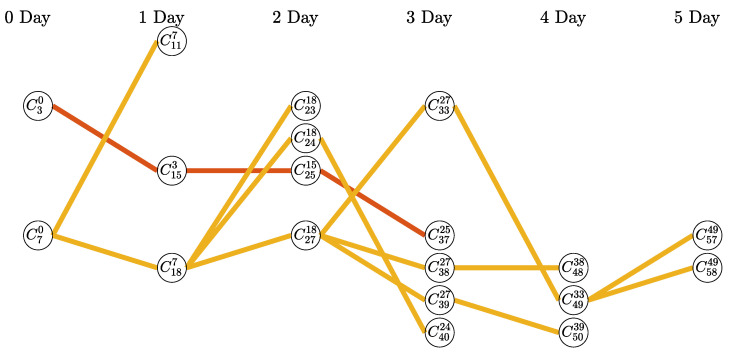
Red chain is a fully ordered set, and the yellow chain is a partially ordered set. Each chain is independent. (For example, it is not possible to compare the order of confirmed patients on the same day in the yellow chain.)

As shown in Figure 4, there are two types of chains: a fully ordered set and a partially ordered set. This is an independent chain because the sources of infection in the two chains are different. The fully ordered set can compare priorities with any two nodes selected; however, the partially ordered set cannot. For example, it is not possible to compare the order of confirmed patients on the same day in the yellow chain shown in Figure 4.

Therefore, if compensation is distributed based on the branches of each node without evenly distributing it within the chain, different compensation may be paid to the confirmed patients on the same day in the case of the partially ordered set. The P2 policy assumes that rewards are evenly distributed within each chain for simplicity of exposure. The compensation for each chain is the same; however, the higher the number of confirmed infected people constituting the chain, the lower the compensation paid to one confirmed person. Voluntary tests of previously unidentified infected people rather than passive large-scale tests involving large-scale medical staff can help reduce the spread of COVID-19 by quickly identifying and isolating the confirmed patients and filtering out the confirmed patients early, which eventually reduces economic losses. The P3 policy is proposed to prevent moral hazards such as intentional infection for rewards. We analyze the compensation paid for each chain for P2 and P3 based on the total compensation *R* of P1. As a simple result, Table 2 presents the compensation that is equally distributed to people in each chain for the infection network shown in Figure 3.

**Figure 5:**
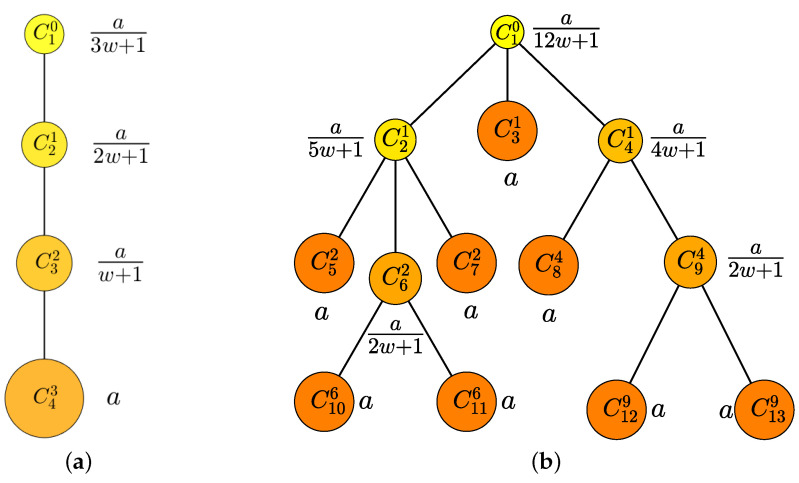
Example of P3: (**a**) Fully ordered set (simple) with $I_{C}=\left\{1,2,3,4\right\}$ and (**b**) Partially ordered set (complex) with $I_{C}=\left\{1,2,\cdots ,\;13\right\}$. The size and color of the node mean the size of the reward. The larger the size and darker the color of the node, the larger the reward to be paid. The rewards for each node that make the sum of the rewards of each chain (**a**,**b**) become $U_{C}$ are expressed using *a* and *w*.

Next, we present a simple example of unevenly paying compensation to people in the chain. During the observation day, the reward $R_{m}$ of the *m*-th infectee is defined as follows, depending on $n_{m}$, which is the number of people infected directly or indirectly by the *m*-th infectee.

(1) $$\begin{array}{c} R_{m}=\frac{a}{wn_{m}+1}, \end{array}$$

where *m* is the number of the infectees and the positive constant *w* is the weight that controls the penalty for further spreading the virus. The sum of the reward for confirmed individuals in one chain must be $U_{C}$. Here, we use the following constraint.

(2) $$\begin{array}{c} U_{C}=\sum_{m\in I_{C}}R_{m}=\sum_{m\in I_{C}}\frac{a}{wn_{m}+1}, \end{array}$$

where $I_{C}$ is a set of infection numbers belonging to the chain, and *a* is a parameter that satisfies the constraint Equation (2). For a chain with a fully ordered set as shown in Figure 5a, $C_{4}^{3}$ is paid $R_{4}=a$ because no one became infected from it, $C_{3}^{2}$ is paid $R_{3}=a/\left(w+1\right)$ because it infected $C_{4}^{3}$, and $C_{2}^{1}$ and $C_{1}^{0}$ are paid $R_{2}=a/\left(2w+1\right)$ and $R_{1}=a/\left(3w+1\right)$, respectively. Figure 5b illustrates the case of a chain with a complex partially ordered set.

## 4. Results

In this section, we present the computational results of estimating the total reward required to control COVID-19 outbreaks using a financial incentive policy.

**Figure 6:**
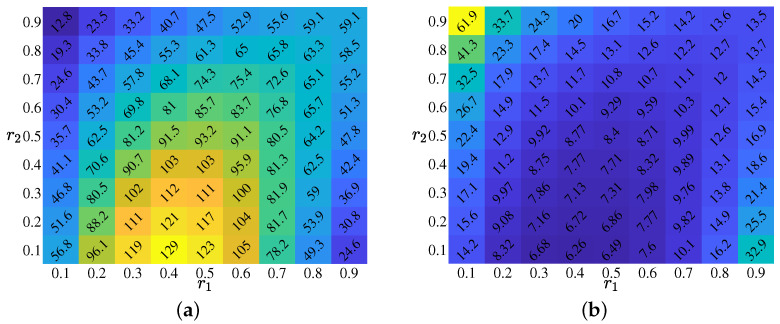
Effect of ratios $r_{1}$ and $r_{2}$: (**a**,**b**) visualize the average number of chains and the average number of confirmed patients that make up the chain according to the combination of $r_{1}$ and $r_{2}$, respectively.

First, to determine the change in the number of chains according to the ratios $r_{1}$ and $r_{2}$, the average number of chains $N_{c}$ corresponding to the combinations $\left(r_{1},r_{2}\right)$ is calculated by increasing the two ratios by 0.1 from 0.1 to 0.9. At this time, 100 simulations were performed for each combination and the number of confirmed people on the *n*-th day C(n)=100 for n=0,1,⋯,7. Figure 6a shows the average number of chains based on the combination of the two ratios $r_{1}$ and $r_{2}$. The number of cells in each cell is presented in Figure 6a, and the mean is $N_{c}$. In a situation where the number of confirmed patients is fixed, the change in the number of chains over a certain period can be interpreted as a change in the number of confirmed cases constituting each chain in a different sense. In each cell of Figure 6b, a number is an average number of confirmed patients constituting a chain, and as $r_{2}$ decreases, the number of members of the chain decreases. This is expected to prevent the spread of the virus.

**Figure 7:**
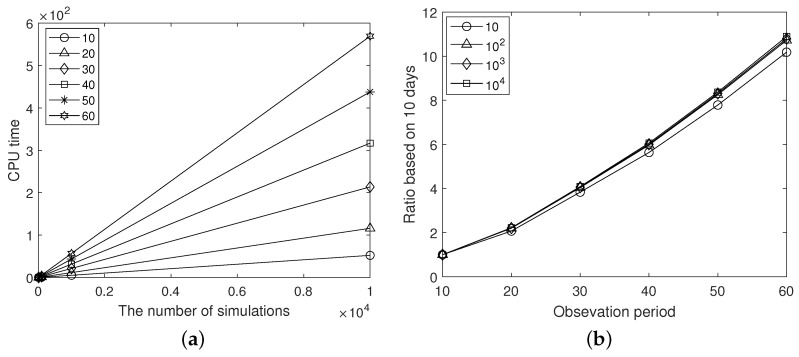
(**a**) Measured CPU time for the observation periods 10,20,⋯,60 and (**b**) for the number of simulations $10,10^{2},\cdots ,\;10^{4}$, the ratio of CPU time based on a 10-day observation period.

Second, in order to find out the complexity of the presented MCS, the CPU time was measured by different observation periods and the number of simulations of the MCS. The simulation setting is the same as the first test condition except for the observation period and the number of simulations. We calculated the average CPU times of 10 cases for the observation periods 10,20,⋯, and 60 days, and the numbers of simulations are $10,\;10^{2},\cdots ,\;$ and $10^{4}$. Here, because of C(n)=100, the longer the observation period is, the higher the total number of confirmed cases. Table 3 and Figure 7 show the computational test results. In parentheses in Table 3, the ratio based on the observation period of 10 days is indicated. As the observation period increases, the number of confirmed cases constituting the infected network increases linearly; however, the CPU time increases relatively quickly. Furthermore, from Figure 7a, we can see that the increase in CPU time according to the number of simulations shows linearity.

**Figure 8:**
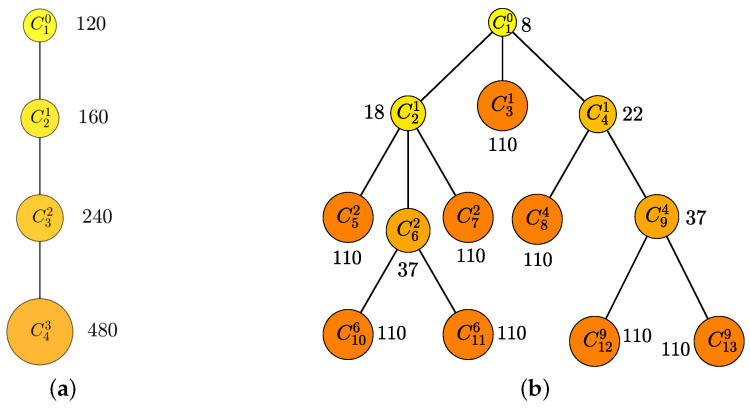
Example of P3: (**a**) the fully ordered set and (**b**) the partially ordered set. The number next to each node is reward $R_{m}$. As a detailed example of Figure 5, when $U_{C}=1000$ and w=1, (**a**) is a=480, and (**b**) is a=110.

Next, if $U_{C}=1000$ and w=1, then a=480 and a=110 for the simple and complex cases, respectively, as shown in Figure 5a,b, respectively. Refer to Figure 8 for a detailed reward at each node.

**Figure 9:**
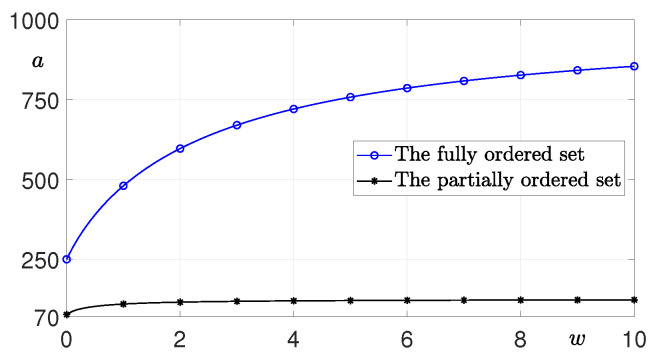
In the case of Figure 8, reward *a* for w=[0,10] with $U_{C}=1000$. When w=0, it implies policy P2. In both chains, when *w* increases, *a* converges to a constant value.

Incentive $R_{m}$ increases as $n_{m}$ decreases. This can be expected to slow the spread of the virus by giving larger rewards to confirmed patients who have directly or indirectly infected a small number. In addition, the effect on weight *w* that adjusts the intensity of the penalty as can be seen in Figure 9 shows the reward *a* of the confirmed people who did not infect a single person for weight *w* with $U_{C}=1000$. Here, w=0 indicates the P2 policy, which is the case of distributing equally to those who belong to each chain. In both chains, the reward *a* converges to a constant value as *w* increases.

## 5. Conclusions

In this study, we present an MCS method for estimating the total cost required to control the spread of the COVID-19 pandemic through financial incentives. One of the greatest difficulties in controlling the spread of the COVID-19 pandemic is that there are infected people who are not yet identified and can transmit the virus to other people. Therefore, it is extremely urgent to rapidly identify and isolate the unidentified infected to avoid further spreading COVID-19. To achieve this purpose, we can consider providing a financial incentive for the confirmed people who voluntarily took the COVID-19 test and tested positive. MCS is used to generate infection network information, which can be found through epidemiological investigations in the simulation. In addition, to prevent the abuse of the financial incentive policy, several conditions should be satisfied in order to receive the incentive. For example, the incentive is offered only if the confirmed people should know from whom they became infected. Thus, we compared and analyzed three policies: a policy (P1) that pays equal compensation to everyone and policies that distribute compensation equally (P2) or unevenly (P3) to people who make up each chain of infection routes. The P2 and P3 policies can be expected to encourage voluntary COVID-19 testing of suspected patients who experience the symptoms and to help control coronavirus transmission. When distributing compensation unevenly, we proposed a policy to allocate smaller compensation by imposing penalties based on the number of people infected directly or indirectly by an infectee and the weight that adjusts the intensity of the penalty. The financial incentive considered for positive patients is financial support for cash types, e.g., gift certificates, etc. To estimate the total cost of incentives, the computational results using MCS based on real epidemiological data, instead of using a mathematical epidemic model for the COVID-19 pandemic disease, can help public health policymakers to implement and use the proposed method to prevent the spread of the COVID-19 pandemic. In addition, the incentive policy is expected to prevent the spread of COVID-19, help prepare for various preparations, e.g., hospital bed preparation, vaccine development, compensate for the gap in economic activities caused by quarantine, and help recover from the economic recession caused by restrictions on business hours and group activities such as social distancing, etc. There are many studies on a mathematical diffusion model that considers vaccination and vaccine effects. Therefore, we will expand our research on policies that encourage vaccine effectiveness or vaccination in the proposed method in future work.

## Figures and Tables

**Table 1:** Parameters of the proposed algorithm.

Symbol	Description of Parameters
$C_{m}^{p}$	Individual-specific information of confirmed COVID-19-positive users
*m*	Infectee number
*p*	The parent number of the *m*-th infectee
C(n)	The number of the confirmed people on the *n*-th day
*N*	Total number of the confirmed people during the observation day (∑C(n))
$I_{known}^{n}$	Set of numbers of confirmed people who know from whom they were infected
	on the *n*-th day
$I_{unknown}^{n}$	Set of numbers of confirmed people who do not know from whom they were infected
	on the *n*-th day
$I^{n}$	Set of identification numbers of confirmed people on the *n*-th day
$N_{n}$	The number of $I_{known}^{n}$
$r_{1}$	Ratio of finding someone who infected them
$r_{2}$	Ratio of being the next generation’s parents in ${\bar{I}}_{known}^{n}$
$I_{spreader}^{n}$	Set of infection spreader number among confirmed people in $I^{n-1}$
$I_{known,spreader}^{n}$	Set of infection spreader number among confirmed people in $I_{known}^{n-1}$
$I_{unknown,spreader}^{n}$	Set of infection spreader number among confirmed people in $I^{n-1}\backslash I_{known}^{n-1}$
${\bar{I}}_{known}^{n}$	Set of confirmed people number by $I_{known,spreader}^{n-1}$
$N_{c}$	The number of chains
$U_{P}$	Unit of reward per person for individual payments
$U_{C}$	Unit of reward per chain for payments by chain
*R*	Total reward for individual payments
$I_{C}$	Set of the infection number belonging to the chain
$n_{m}$	The number of people infected directly or indirectly from the *m*-th infectee
$R_{m}$	Reward of the *m*-th infectee
*w*	Weight of control the intensity of the penalty for spreading virus
*a*	Reward for a confirmed person who did not infect in the chain

**Table 2:** Compensation equally distributed to people in each chain in the infection network as shown in Figure 3. The compensation is rounded off to the first decimal place, and the numbers in parentheses are the number of people in each chain.

Policy	Chain 1	Chain 2	Chain 3	Chain 4	Chain 5	Chain 6	Chain 7
P1	100	100	100	100	100	100	100
P2	71 (14)	250 (4)	67 (15)	200 (5)	333 (3)	83 (12)	200 (5)

**Table 3:** CPU times (in seconds) for the presented algorithm with the observation periods are 10,20,⋯, and 60 days, and the numbers of simulations are $10,\;10^{2},\cdots ,\;$ and $10^{4}$.

	10	20	30	40	50	60
$10^{1}$	0.0561 (1.0)	0.1163 (2.1)	0.2154 (3.8)	0.3163 (5.6)	0.4371 (7.8)	0.5717 (10.2)
$10^{2}$	0.5295 (1.0)	1.1656 (2.2)	2.1411 (4.0)	3.1617 (6.0)	4.3713 (8.3)	5.6781 (10.7)
$10^{3}$	5.2617 (1.0)	11.6098 (2.2)	21.3773 (4.1)	31.6089 (6.0)	43.7265 (8.3)	56.7887 (10.8)
$10^{4}$	52.2478 (1.0)	116.1130 (2.2)	213.6878 (4.1)	316.8556 (6.1)	437.5807 (8.4)	569.1442 (10.9)

## Data Availability

Not applicable.

## Appendix A. MATLAB Code of Incentive

Table A1 presents the reward for $n_{m}$ for each chain with w=1 for the infection network shown in Figure 3. We provide the MATLAB code for the simulation of Table A1 from the website of the corresponding author: https://mathematicians.korea.ac.kr/cfdkim/open-source-codes/ (accessed on 27 November 2022).

**Table A1:** In the infection network shown in Figure 3, the reward for $n_{m}$ of each chain was presented rounding off to the first decimal place with w=1, and the numbers in parentheses are the number of people receiving the corresponding reward.

$n_{m}$	Chain 1	Chain 2	Chain 3	Chain 4	Chain 5	Chain 6	Chain 7
0	143 (4)	480 (1)	107 (7)	270 (3)	429 (2)	113 (8)	270 (3)
1	71 (3)	240 (1)	54 (3)	135 (1)	0 (0)	56 (1)	135 (1)
2	48 (3)	160 (1)	36 (1)	0 (0)	143 (1)	0 (0)	0 (0)
3	0 (0)	120 (1)	27 (1)	0 (0)	0 (0)	0 (0)	0 (0)
4	29 (1)	0 (0)	0 (0)	54 (1)	0 (0)	0 (0)	54 (1)
5	24 (1)	0 (0)	0 (0)	0 (0)	0 (0)	19 (1)	0 (0)
6	0 (0)	0 (0)	0 (0)	0 (0)	0 (0)	0 (0)	0 (0)
7	0 (0)	0 (0)	0 (0)	0 (0)	0 (0)	0 (0)	0 (0)
8	0 (0)	0 (0)	12 (1)	0 (0)	0 (0)	0 (0)	0 (0)
9	0 (0)	0 (0)	0 (0)	0 (0)	0 (0)	11 (1)	0 (0)
10	0 (0)	0 (0)	0 (0)	0 (0)	0 (0)	0 (0)	0 (0)
11	0 (0)	0 (0)	0 (0)	0 (0)	0 (0)	9 (1)	0 (0)
12	11 (1)	0 (0)	8 (1)	0 (0)	0 (0)	0 (0)	0 (0)
13	10 (1)	0 (0)	0 (0)	0 (0)	0 (0)	0 (0)	0 (0)
14	0 (0)	0 (0)	7 (1)	0 (0)	0 (0)	0 (0)	0 (0)

The main code is as follows:
